# Class I Polyhydroxyalkanoate (PHA) Synthase Increased Polylactic Acid Production in Engineered Escherichia Coli

**DOI:** 10.3389/fbioe.2022.919969

**Published:** 2022-06-23

**Authors:** Mengxun Shi, Mengdi Li, Anran Yang, Xue Miao, Liu Yang, Jagroop Pandhal, Huibin Zou

**Affiliations:** ^1^ State Key Laboratory Base of Eco-Chemical Engineering, College of Chemical Engineering, Qingdao University of Science and Technology, Qingdao, China; ^2^ Department of Chemical and Biological Engineering, The University of Sheffield, Sheffield, United Kingdom; ^3^ CAS Key Laboratory of Bio-based Materials, Qingdao Institute of Bioenergy and Bioprocess Technology, Chinese Academy of Sciences, Qingdao, China

**Keywords:** class I polyhydroxyalkanoate synthase, engineered *E. coli*, polylactic acid, biopolyester, degradable polymer

## Abstract

Polylactic acid (PLA), a homopolymer of lactic acid (LA), is a bio-derived, biocompatible, and biodegradable polyester. The evolved class II PHA synthase (PhaC1_*Ps*6-19_) was commonly utilized in the *de novo* biosynthesis of PLA from biomass. This study tested alternative class I PHA synthase (PhaC_*Cs*_) from *Chromobacterium* sp. USM2 in engineered *Escherichia coli* for the *de novo* biosynthesis of PLA from glucose. The results indicated that PhaC_*Cs*_ had better performance in PLA production than that of class II synthase PhaC1_*Ps*6-19_. In addition, the *sulA* gene was engineered in PLA-producing strains for morphological engineering. The morphologically engineered strains present increased PLA production. This study also tested fused propionyl-CoA transferase and lactate dehydrogenase A (fused Pct_*Cp*_/LdhA) in engineered *E. coli* and found that fused Pct_*Cp*_/LdhA did not apparently improve the PLA production. After systematic engineering, the highest PLA production was achieved by *E. coli* MS6 (with *PhaC*_*Cs*_ and *sulA*), which could produce up to 955.0 mg/L of PLA in fed-batch fermentation with the cell dry weights of 2.23%, and the average molecular weight of produced PLA could reach 21,000 Da.

## Introduction

Biopolyesters have been developed in recent years as alternatives to petroleum-based synthetic polyesters (Nduko and Taguchi, 2021). As a commercialized biopolyester, polylactic acid (PLA) can be prepared from renewable biomass with promising physical performance, biocompatibility, and biodegradability (Taguchi et al., 2008; Yang et al., 2010; Paço et al., 2019). The industrial preparation of PLA is accomplished through several steps: first, the monomer of lactic acid (LA) is prepared from biomass through fermentation; then LA is converted to lactide followed by the ring-opening polymerization of lactide to PLA (Maharana et al., 2009).

With the fast development of biotechnology, PLA homopolymer and LA-containing copolymers can be *de novo* biosynthesized from renewable biomass by engineered strains (Taguchi et al., 2008; Yang et al., 2010; Choi et al., 2016; Zou et al., 2021). However, compared with the efficient bioproduction of LA-containing copolymers, the microbial production of PLA homopolymers is still challenging with low productivity. One of the barriers in the biosynthesis of the PLA homopolymer is that the PHA synthases involved exhibit higher activities toward other substrates than LA monomer (Park et al., 2012).

Based on the primary structure, subunit compositions, and substrate specificity, four classes of PHA synthases have been found in nature (Yang et al., 2010; Zou et al., 2017; Chek et al., 2019). Class I, III, and IV PHA synthases prefer short-chain length (SCL) monomers, whereas class II PHA synthases exhibit higher activities toward medium-chain length (MCL) monomers (Rehm, 2003; Tsuge et al., 2015; Zou et al., 2017). Class II synthase from *Pseudomonas* sp. was engineered (E130D, S325T, S477G, and Q481K) to gain the ability to synthesize PLA and LA-containing copolymers. Although the engineered strains (with engineered class II synthase) can efficiently produce LA-containing copolymers (Jung et al., 2010; Yang et al., 2010; Choi et al., 2016; Li et al., 2016), PLA homopolymer productivity was as low as 0.5 wt% of dry cell weight (Yang et al., 2010; Park et al., 2012). The low productivity of the PLA homopolymer was presumably due to the low substrate specificity of PHA synthase (Park et al., 2012), low mobility of the generated PLA, and low concentration of LA-CoA in the engineered strains (Matsumoto et al., 2018). Robust PHA synthases with higher substrate specificity toward LA monomer need to be discovered or engineered for the enhancement of the biosynthesis of PLA homopolymers (Taguchi and Matsumoto, 2021).

Morphological engineering is another trend in the biosynthesis of biopolymers, which aims at larger cell space for the storage of the produced biopolymers *in vivo*. FtsZ, a bacterial tubulin homolog, is one of the targets in the morphological engineering of bacteria strains (Erickson et al., 2010; Chen et al., 2012; Wang et al., 2014). The inhibition of FtsZ can affect the formation of the Z ring during bacteria division and hence enlarge cell space (Dajkovic et al., 2008; Adams and Errington, 2009; Chen et al., 2012). Higher expression of SulA can inhibit FtsZ and reduce the cell division rate (Dajkovic et al., 2008; Chen et al., 2012). For example, morphologically engineered *E. coli* JM109 exhibits the increased production of poly (3-hydroxybutyrate) (PHB) (from 8 to 9 g/L) and poly (3HB-co-4HB) (from 8.2 to 9.2 g/L) (Wang et al., 2014; Wu et al., 2016).

In this study, to screen robust PHA synthase in PLA biosynthesis, we selected the class I PHA synthase from *Chromobacterium* sp. USM2, which has been used in the polymerization of 3-hydroxypropionic acid (3HP, an isomer of LA) (Linares-Pastén et al., 2015), and evaluated its performance in PLA homopolymer production by engineered *E. coli*. In addition, we additionally expressed the *sulA* gene and evaluated the PLA production of morphologically engineered strains.

## Materials and Methods

### Strains and Engineering Methods

All the information on strains and plasmids is listed in Table 1. The information of key plasmids is provided in Supplemental Figure S1. The primers are summarized in Supplemental Table S1. Engineered strains were constructed using the following methods.

**TABLE 1:** Strains and plasmids used in this study.

Names	Description	Reference/Source
Strains		
*E. coli* χ7213	Donor strain for gene deletion	Laboratory-stored
*E. coli* MS1	*△ackA* from *E. coli* BL21	This study
*E. coli* MS2	Express *ldhA* gene in *E. coli* MS1	This study
*E. coli* MS3	Express the evolved Pct_*Cp*_ gene and the evolved *PhaC1*_*Ps*6-19_ gene in *E. coli* MS2	This study
*E. coli* MS4	Express *sulA* gene in *E. coli* MS3	This study
*E. coli* MS5	Express the evolved Pct_*Cp*_ gene and the evolved *PhaC1*_*Cs*_ gene in *E. coli* MS2	This study
*E. coli* MS6	Express *sulA* gene in *E. coli* MS5	This study
*E. coli* MS7	Express the gene of fused enzyme of Pct_*Cp*_/LdhA, the evolved *PhaC1*_*Ps*6-19_ gene, and *sulA* gene in *E. coli* MS1	This study
Plasmids		
pTrcHis2B	Ap^R^	Laboratory-stored
pACYCDuet-1	Cm^R^	Laboratory-stored
pET30a	Kan^R^	Laboratory-stored
pRE112-△ackA	Cm^R^	This study
ldhA-pTrcHis2B	*ldhA* from *E. coli* inserted into the pTrcHis2B vector under trc promoter	This study
ldhA-sulA-pTrcHis2B	*ldhA* from *E. coli* BL21 and *sulA* from *E. coli* str. K-12 inserted into the pTrcHis2B vector under trc promoter	This study
Pct_*Cp*_-pACYCDuet-1	Evolved Pct_*Cp*_ (V193A) inserted into the pACYCDuet-1 vector under T7 promoters	This study
Pct_*Cp*_-PhaC_*Ps*6-19_-pACYCDuet-1	Evolved Pct_*Cp*_ (V193A) and evolved PhaC1_*Ps*6-19_ (E130D, S325T, S477G, and Q481K) inserted into the pACYCDuet-1 vector under T7 promoters	This study
Fused-Pct/ldhA-pET30a	LdhA and Pct fusion enzyme under flexible linker (GGGGS)_3_	This study
Pct_*cp*_-PhaC_*Cs*_-pACYCDuet-1	Evolved *Pct*_*Cp*_ (V193A) and *PhaC1*_*Cs*_ inserted into the pACYCDuet-1 vector under T7 promoters	This study

Ap^R^, ampicillin resistance; Cm^R^,chloramphemicol resistance; Kan^R^, kanamycin resistance.

The suicide plasmid–mediated genome editing method (Gao et al., 2014) was utilized in the deletion of the *ackA* gene from the genome of *E. coli* BL21 (DE3). The homolog arms (600 bp upstream and downstream of the *ackA* gene) were cloned into the suicide plasmid of pRE112*.* The constructed pRE112-*△ackA* was transformed into *E. coli χ*7213 to construct the donor strain of *E. coli χ*7213/pRE112-*△ackA*. Then, the conjugation occurred between the donor strain and the receptor strain of *E. coli* BL21 (DE3) for the deletion of the *ackA* gene. The strain of *E. coli* MS1 (Table 1) was constructed after knocking off *ackA*.

**FIGURE 1:**
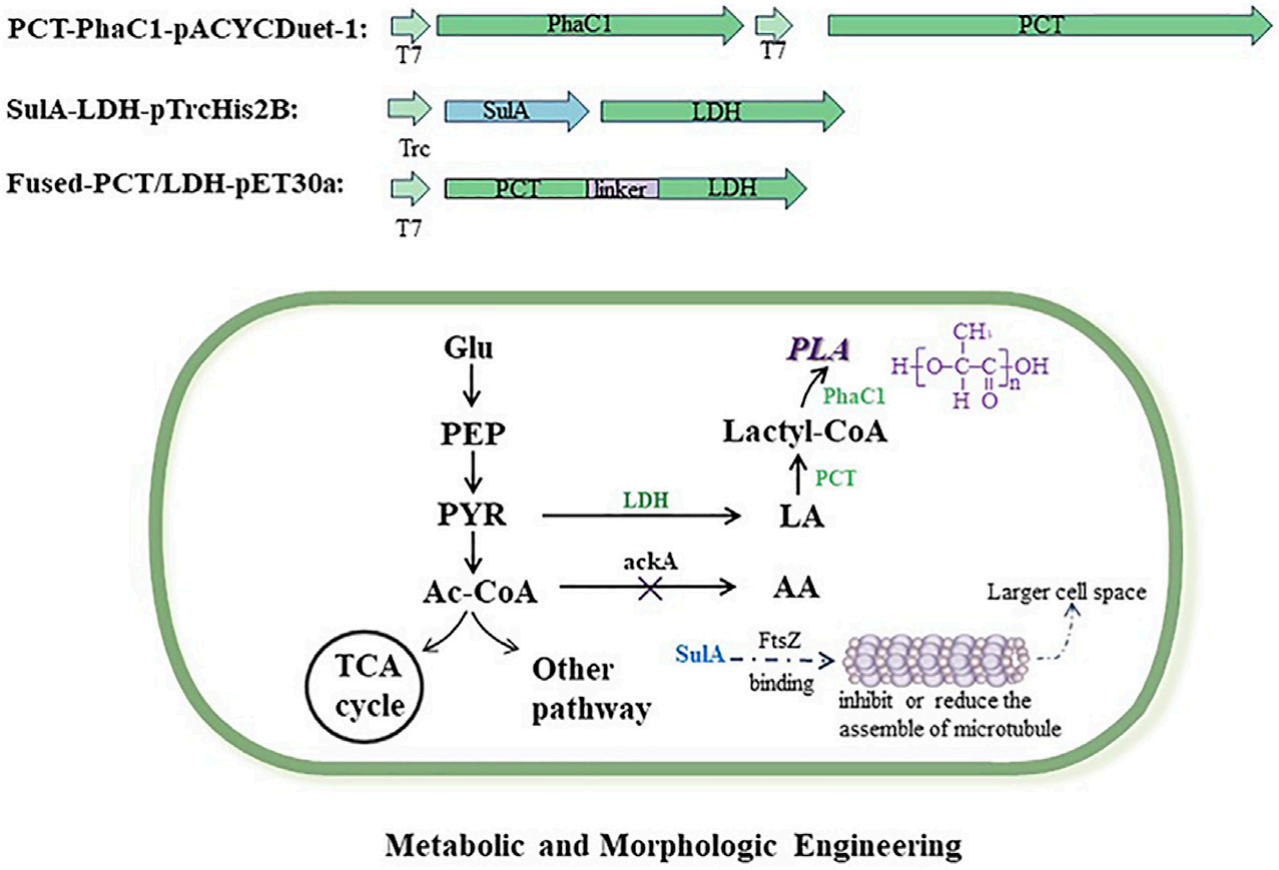
Metabolic and morphologic engineering of *E. coli* strains for polylactic acid production. Exogenous vectors expressed the genes of key enzymes which are involved in the biosynthesis pathway of polylactic acid. The gene of *ackA* was deleted in the engineered strains to reduce the competitive by-product of acetic acid. The gene of *sulA* was expressed in engineered stains for larger cell space. Full names of abbreviations are as follows: PLA, polylactic acid; PhaC, PHA synthase; PCT, propionyl-CoA transferase; LDH, lactate dehydrogenase; Glu, glucose; PEP, phosphoenolpyruvate; PYR, pyruvate; Ac-CoA, acetyl co-enzyme A; AA, acetic acid; LA, lactic acid.

Based on the chassis strain of *E. coli* MS1, other engineered strains (MS2–MS7) were constructed using the following methods. As shown in Figure 1, the biosynthetic pathway of PLA was engineered in PLA-producing strains; in addition, the *sulA* gene was expressed in strains for larger cell space.

From *E. coli* MS1, *E. coli* MS2 additionally expressed the *ldhA* gene from *E. coli* BL21 (lactate dehydrogenase, NCBI No. NC_012892.2). From *E. coli* MS2, *E. coli* MS3 additionally expressed the evolved *Pct*_*Cp*_ gene (propionyl-CoA transferase, NCBI No. CAB77207.1, with mutation of V193A) from *Clostridium propionicum* DSM 1682 and the evolved *PhaC1*_*Ps*6-19_ gene (class II PHA synthase, NCBI No. ACM68707.1, with mutations of E130D, S325T, S477G, and Q481K) from *Pseudomonas* sp. MBEL 6-19. From *E. coli* MS3, *E. coli* MS4 additionally expressed the *sulA* gene from *E. coli* str. K-12 substr. MG1655 (NCBI No. NC_000913.3).

From *E. coli* MS2, *E. coli* MS5 additionally expressed the evolved *Pct*_*Cp*_ gene from *C. propionicum* and the *PhaC*_*Cs*_ gene (class I PHA synthase, NCBI No. ADL70203.1) from *Chromobacterium* sp. USM2. From *E. coli* MS5, *E. coli* MS6 additionally expressed the *sulA* gene from *E. coli* str. K-12.

From *E. coli* MS1, *E. coli* MS7 additionally expressed the gene of the fused enzyme of Pct_*Cp*_/LdhA (Supplemental Figure S2). A flexible linker (Gly–Gly–Gly–Gly–Ser)_3_ was inserted between Pct_*Cp*_ and LdhA; in addition, *E. coli* MS7 expressed the evolved *PhaC1*_*Ps*6-19_ gene from *C. propionicum* and the *sulA* gene from *E. coli* str. K-12.

### Flask and Fed-Batch Fermentation

All strains utilized in this study are cultivated in the Luria–Bertani (LB) medium (flask-level) or M9 medium (fermenter-level). The LB medium contains (per liter) 10 g tryptone, 5 g yeast extract, and 10 g NaCl. The M9 medium consists of (per liter) 1 g (NH_4_)_2_SO_4_, 3 g K_2_HPO_4_.3H_2_O, 1.9 g KCl, 5 g yeast extract, 1 g sodium citrate, 1 g citric acid, 1 g glycine betaine, 0.24 g MgSO_4,_ and 1 ml of the stored solution of trace element. The stored solution of trace element consists of (per liter) 3.7 g (NH_4_)_6_Mo_7_O_24_·4H_2_O, 2.9 g ZnSO_4_ 7H_2_O, 24.7 g H_3_BO_3_, 2.5 g CuSO_4_ 5H_2_O, and 15.8 g MnCl_2_ 4H_2_O. Variable antibiotics were supplemented in the cultivation medium for different strains (Table 1). The concentration of antibiotics in the medium is as follows: ampicillin 48 mg/L, chloramphenicol 24 mg/L, and kanamycin 45 mg/L. The sucrose-containing (10% w/w) LB medium was utilized to screen the strain of *E. coli* MS1.

For fermentation of PLA, individual strains were cultivated in 10 ml LB medium at 37°C overnight in a rotary shaker at 220 rpm. Then, 5% (V/V) seed cultures were added into 100 ml LB medium (in a flask) and cultivated at 37°C overnight in a rotary shaker at 220 rpm. The secondary seed cultures were inoculated into a 5-L fermenter (Bailun Inc., China) containing 2 L M9 medium, and 20 g/L glucose was added as a starting carbon source. Fed-batch fermentation starts at 37°C. During the fermentation process, ammonium hydroxide solution (6M) was automatically added to maintain the pH at 7. The dissolved oxygen concentration (DOC) was maintained at 10–20% by changing the agitation speed and ventilatory capacity (VC). Ten hours after inoculation, 0.5 mM of isopropyl-*β*-d-thiogalactopyranoside (IPTG) was added in the fermenter, and then the cultivation temperature was decreased to 30°C. Then, 100 ml of 50% glucose (w/v) was supplemented every 12 h. The cell growth value of OD_600_ was monitored using the spectrophotometer at 600 nm. The duration time of fermentation was 72 h.

### Analytical Methods

The solvent extraction method (Jung et al., 2010) was utilized for the purification of PLA products from the cells. After fermentation, the cells were harvested by centrifugation at 4000 rpm for 20 min. The harvested cells were washed twice with absolute ethanol and distilled water before lyophilization. Then, the cells were lyophilized for 24 h and the cell dry weights (CDW) of different samples were measured and recorded. PLA was extracted from dried cells by chloroform in the Soxhlet apparatus at 80°C for 16 h. Excessive chloroform was removed using a rotary evaporator, and cell debris was removed by passing through a PTFE filter. PLA was precipitated by adding five-fold ice-cold methanol. Weights of purified and dried PLA was measured and recorded.

To qualitatively determine the polymer structure, the samples were analyzed by ^1^H and ^13^C nuclear magnetic resonance (NMR) spectra using a Bruker AM-500 MHz spectrometer at 500 and 125 MHz, respectively. The sample was solved in CDCl_3_ with tetramethylsilane (TMS) as an internal chemical shift standard. The number-average molecular weight (*M*_*n*_) and the weight-average molecular weight (*M*_*w*_) of PLA were determined by gel permeation chromatography (GPC) equipped with TSKgel SuperMutipore HZ-M*2 column and GPC data processing software. The PLA sample (1 mg/ml) was eluted using tetrahydrofuran (THF) before injection (20 μl), and GPC was operated at a flow rate of 0.35 ml/min at 40°C. Polystyrene with a narrow range of polydispersity (1.03-1.05) was used for calibration.

The morphological form of engineered *E. coli* was characterized using a scanning electron microscope (SEM) on a Hitachi S-4800 instrument. The cells were harvested by centrifugation at 5000 rpm for 5 min and subsequently washed with phosphate-buffered saline (PBS) (pH 7.4) three times. Then, the washed cells were fixed with 2.5% (V/V) glutaraldehyde overnight at 4°C. The fixed cells were washed again with phosphate-buffered saline (PBS) (pH 7.4) three times (30 min each). Afterward, ethanol gradients of 30%, 50%, 70%, 80%, 90%, and 95% (V/V) solutions were used to dehydrate the fixed cells in a sequential way. The cell samples were further dehydrated with 100% ethanol three times. After that, tertiary butyl alcohol was mixed with ethanol in a ratio of 1:1 and pure tertiary butyl alcohol was used to achieve metathesis of ethanol in the cells (Wang et al., 2014). At last, the cells were mixed in with tertiary butyl alcohol and lyophilized for imaging.

The PLA granules in *E. coli* cells were characterized using a transmission electron microscope (TEM) on a Hitachi H-7650 instrument. The cells were harvested by centrifugation at 5000 rpm for 5 min and subsequently washed with phosphate-buffered saline (PBS) (pH 7.4) three times. Then, the washed cells were fixed with 2.5% (V/V) glutaraldehyde overnight at 4°C. The fixed cells were washed again with phosphate-buffered saline (PBS) (pH 7.4) three times (30 min each), and then 1% (V/V) osmic acid was added to fix the cell for 1 h. The fixed cells were washed again with PBS (pH 7.4) three times (30 min each). Afterward, acetone gradient solutions of 30%, 50%, 70%, 80%, 90%, and 95% (V/V) were used to dehydrate the fixed cells in a sequential way. The resulting cell samples were further dehydrated with 100% acetone three times. Finally, the cell samples are embedded with Spurr resin for imaging.

### Statistical Methods

The significance of differences between the mean values of testing samples was compared using Student’s t-test. Differences were considered statistically obvious if *p* < 0.05 and significant if *p* < 0.01.

## Results and Discussion

### Biosynthesis of PLA *via* Class I PHA Synthase (From *Chromobacterium* sp. USM2)

**FIGURE 2:**
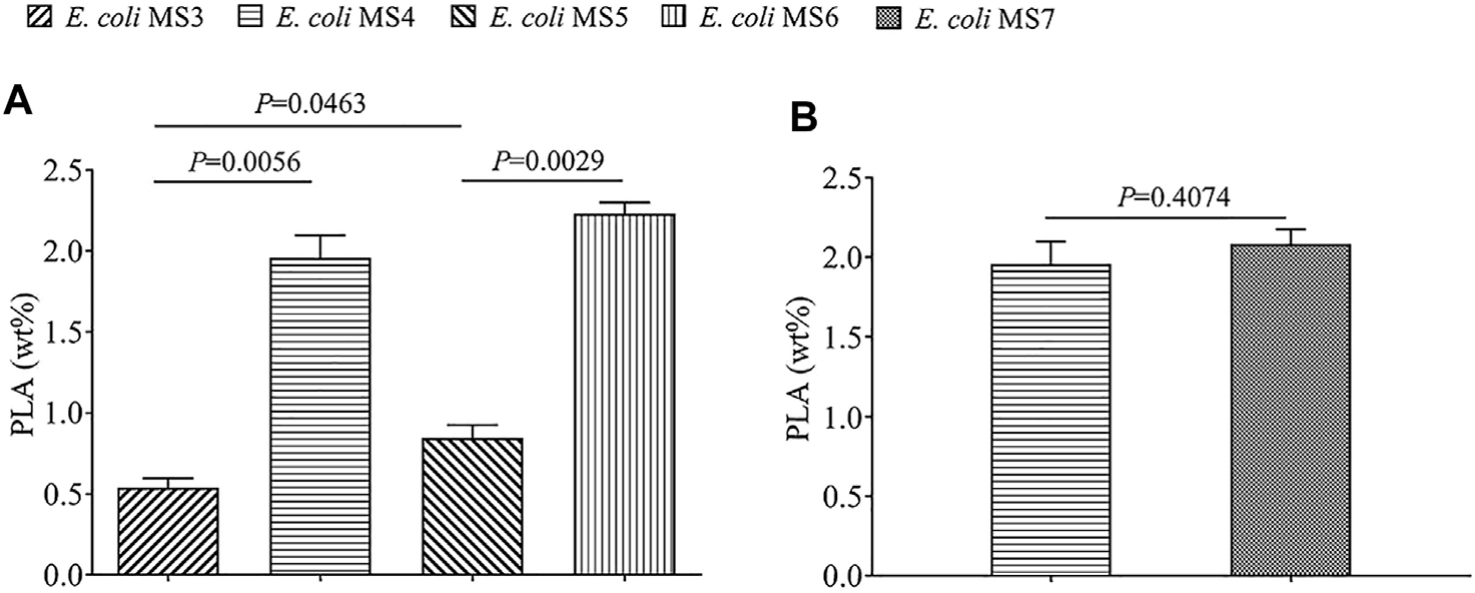
Comparison of the PLA production by five different engineered strains. The PLA production was recorded by wt% (weight of PLA/dry cell weight). The data shown are expressed as means of three repeated experiments, and the error bars present their standard deviation. Statistical difference (*p* value) between different groups is also present. **(A)** Strains which express the gene of PhaC1_*Cs*_ (class I PHA synthase) and *sulA* (for morphological engineering) have higher PLA production. **(B)** Strain which expresses the fused enzyme of Pct_*Cp*_/LdhA has similar PLA production with the control strain.

The qualitative ^1^H and ^13^C assay of polymer product (Supplemental Figure S3) confirmed that PLA homopolymer was *de novo–*biosynthesized from glucose by the engineered strains of *E. coli*, and the ^1^H assay also indicated the presence of oligomeric PLA or LA monomer indicated the hydrolysis of PLA during analysis processing. The quantitative comparison of PLA production by different strains (Figure 2) showed that *E. coli* MS5 (with *PhaCcs*) could produce up to 323.4 mg/L of PLA (0.85% of CDW), obviously higher (*p* < 0.05) than the PLA production (189.5 mg/L, 0.54% of CDW) by the control strain of *E. coli* MS3 (with evolved *PhaC1*_*Ps*6-19_). The results indicated that class I PHA synthase (from *Chromobacterium* sp. USM2) exhibited better performance in the biosynthesis of the PLA homopolymer. This study further compared the *M*_*w*_ of the produced PLA with the strains of MS5 and MS3 (Table 2). Although the PLA products with two strains present a similar average *M*_*w*_ (around 21,000 Da), the lower *M*_*w*_/*M*_*n*_ value of MS5 indicated that the PLA produced with MS5 has more centralized molecular weight distribution than that of MS3.

**TABLE 2:** Gel permeation chromatography (GPC) of PLA synthesized by strains with different PHA synthases.

PHA synthase	*M*_*n*_ ^a^ (Da)	*M*_*w*_ ^b^ (Da)	*M*_*w*_/*M*_*n*_ ^c^
PhaC1_*Ps*6-19_	10,642	23,436	2.202
PhaC_*Cs*_	16,515	21,088	1.277

a *M*_*n*_: number-average molecular weight.

b *M*_*w*_: weight-average molecular weight.

c *M*_*w*_/*M*_*n*_: molecular weight dispersion index, denotes the molecular weight distribution width of the polymer.

As described in earlier studies, only engineered class II PHA synthases were utilized in the biosynthesis of PLA homopolymer and LA-containing copolymers, and the mutation of several residues (E130D, S325T, S477G, and Q481K) can improve their substrate specificities toward LA monomers (Taguchi et al., 2008; Yamada et al., 2009; Jung et al., 2010; Yang et al., 2010; Choi et al., 2016). Class I PHA synthases present higher substrate specificity toward SCL monomers (Zou et al., 2017), but have not been utilized in the biosynthesis of the PLA homopolymer and LA-containing copolymers before. The results of this study confirmed that class I PHA synthase could also be utilized in PLA production (Figure 2). Moreover, the same synthase (PhaC_*Cs*_) could catalyze the polymerization of 3-hydroxypropionic acid (3HP, an isomer of LA), and the CDW of the produced P (3HP) can reach 40% (Linares-Pastén et al., 2015), indicating that class I PhaC_*Cs*_ shows promising substrate specificity toward the SCL hydroxypropionic acids.

### Increased PLA Production by Morphologically Engineered *E. coli*

**FIGURE 3:**
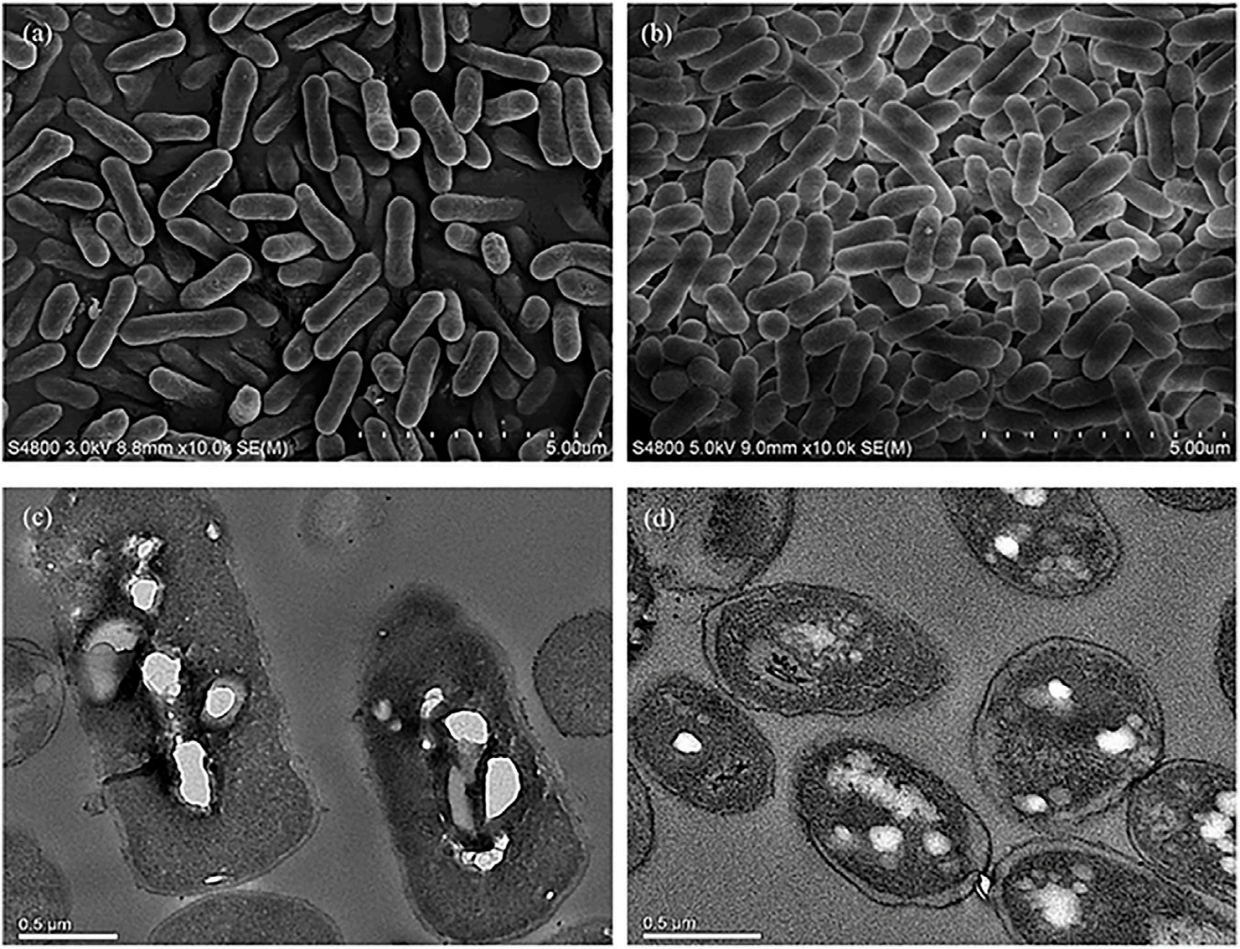
Morphological comparison of strains with or without morphological engineering using SEM and TEM assays. **(A)** SEM assay of cells from the morphologically engineered strain (with the expression of *sulA*). **(B)** SEM assay of cells from the control strain without expressing *sulA*. **(C)** TEM assay of cells from the morphologically engineered strain (with the expression of *sulA*). **(D)** TEM assay of cells from the control strain without expressing *sulA*.

At the time point of 36 h after inoculation, the cells of *E. coli* MS4 (morphological engineering via *sulA*) and MS3 (control strain without *sulA*) were harvested for SEM/TEM analysis. The SEM results revealed that *E. coli* MS4 has an elongated rod cell shape (Figure 3A), which presents a longer cell shape than the control strain of *E. coli* MS3 (Figure 3B). Moreover, TEM results showed that the intracellular PLA granules in *E. coli* MS4 (Figure 3C) occupied larger cell space than in the control strain of *E. coli* MS3 (Figure 3D), indicating the increased PLA production in *E. coli* MS4. The results of fed-batch fermentation (Figure 2) confirmed that *E. coli* MS4 significantly (*p* < 0.01) had higher production of PLA (CDW of 1.96%) than *E. coli* MS3 (CDW of 0.54%). The highest PLA production was achieved by *E. coli* MS6 (contains both *PhaC*_*Cs*_ and *sulA*), which can produce up to 955.0 mg/L of PLA in fed-batch fermentation with the CDW of 2.23%.

The significantly increased PLA production in *E. coli* MS4 and MS6 than their control strains of *E. coli* MS3 and MS5 (Figure 2) indicated that the overexpression of the *sulA* gene in *E. coli* strains could morphologically affect the strains to achieve improved production of intracellular polymers. Similar to this study, *E. coli* strains have been morphologically engineered to achieve the increased production of poly (3-hydroxybutyrate-co-4-hydroxybutyrate) (Wang et al., 2014). It has been demonstrated that SulA could bind the tubulin of FtsZ to inhibit the formation of Z loop in the cell division of *E. coli* strains (Pichoff and Lutkenhaus, 2002, 2005; Chen et al., 2012). The SulA/FtsZ interaction was also found in *Pseudomonas* (Chen et al., 2012), indicating that this strategy can be more broadly applied in the biosynthesis of biopolymers by variable chassis strains.

### Fused Pct_*Cp*_/LdhA Did Not Increase the PLA Production

As shown in Figure 1, LdhA and Pct_*Cp*_ are two key enzymes in the biosynthetic pathway of lactyl-CoA. In order to supply the increased level of lactyl-CoA for PLA biosynthesis, the fused Pct_*Cp*_/LdhA enzyme was engineered in *E. coli* MS7 (Supplemental Figure S2). Compared with the PLA production of the control strain of *E. coli* MS4 (CDW of 1.96%), the PLA production of *E. coli* MS7 (CDW of 2.08%) was not apparently increased (*p* > 0.05). In addition, *E. coli* MS7 (containing Pct_*Cp*_ and LdhA fusion enzyme) had a lower dry cell weight (28.9 g/L, 72 h) than *E. coli* MS4 (31.6 g/L, 72 h), which indicated that the presence of fused enzyme exhibits growth stress toward the engineered strain of MS7.

Similar to other fused proteins and enzymes (Yu et al., 2015), the tandem fusion of Pct_*Cp*_ and LdhA can spatially restrain multiple catalytic domains in one fused enzyme. The results of this study indicated that fused Pct_*Cp*_/LdhA enzyme retained the biocatalytic functions of individual Pct_*Cp*_ and LdhA. However, fused Pct_*Cp*_/LdhA did not apparently improve the general biosynthesis of PLA, which indicated that the final step (polymerization of lactyl-CoA into PLA by PHA synthase) could be the bottleneck step in the biosynthesis of the PLA homopolymer, as reported by earlier studies (Park et al., 2012; Matsumoto et al., 2018).

## Conclusion

The present study demonstrated that class I PHA synthase from *Chromobacterium* sp. USM2 (PhaC_*Cs*_) is feasible to catalyze the polymerization of the PLA homopolymer from lactyl-CoA. In *de novo* PLA fermentation from glucose, engineered *E. coli* strains having PhaC_*Cs*_ present improved PLA production than control strains, which expressed the evolved class II PHA synthase *PhaC1*_*Ps*6-19_. In addition, *sulA*-mediated morphological engineering could enlarge the cell space and further improve PLA production of the engineered *E. coli* strains.

## Data Availability

The original contributions presented in the study are included in the article/Supplementary Material; further inquiries can be directed to the corresponding author.
