# Supplementary material for: Class I Polyhydroxyalkanoate (PHA) Synthase Increased Polylactic Acid Production in Engineered Escherichia Coli
Source: Front Bioeng Biotechnol. 2022 Jun 23;10:919969. doi: 10.3389/fbioe.2022.919969 (PMC9261260; doi:10.3389/fbioe.2022.919969)
Supplement: Supplementary file 1 [file DataSheet1.docx]

***Class I polyhydroxyalkanoate synthase increased polylactic acid production in engineered Escherichia coli***

**Mengxun Shi^1,3^, Mengdi Li^1^, Anran Yang^1^, Xue Miao^1^, Liu Yang^1^, Jagroop Pandhal^3^, Huibin Zou^1,2*^**

^1^State Key Laboratory Base of Eco-Chemical Engineering, College of Chemical Engineering, Qingdao University of Science and Technology, Qingdao, China

^2^CAS Key Laboratory of Bio-based Materials, Qingdao Institute of Bioenergy and Bioprocess Technology, Chinese Academy of Sciences, Qingdao, China

^3^Department of Chemical and Biological Engineering, the University of Sheffield, Mappin Street, Sheffield, United Kingdom

***Correspondence:**

No. 53 Zhengzhou Road, College of Chemical Engineering, Qingdao University of Science and Technology, Qingdao, China 266042. Tel: +86 532 84022879.

Email address: zouhb@qibebt.ac.cn, huibinzou@hotmail.com (H. Zou)

ORCID: 0000-0002-0805-3022 (H. Zou)

# Supplementary Figures and Tables

## Supplementary Table

## Supplementary Table 1. Primers used in this study

| Primers | Sequence (5’-3’) |
| --- | --- |
| ldhA-F | ATGAAACTCGCCGTTTATAGCACAA |
| ldhA-R | CGGCAAGATTAAACCAGTTCGT |
| pTrcHis2B-F | CCGAACGAACTGGTTTAGAACAAAAACTCATCTCAGAAGA |
| pTrcHis2B-R | ATAAACGGCGAGTTTCATGGTTTATTCCTCCTTATTTA |
| ID-ldhA-pTrcHis2B-F | AAGGAGGAATAAACCATG |
| ID-ldhA-pTrcHis2B-R | GGCGCTATTCAGATCCTCTTCT |
| Fused-F | TCATCATTCTTCTGGTCTGGTGCCAATGCGCAAAGTGCCGATTATTACCG |
| Fused-R1 | GCTGCCACCGCCACCTGATTTCATTTCTTTCAGGCCCATC |
| Fused-R2 | AGAACCGCCGCCACC GCTGCCACCACCGCC GCTGCCACCGCCACC |
| Fused-R3 | TGGTGCTGTACACCGCCAGCTTCATAGAACCGCCGCCACC |
| Fused-R4 | CAACTCAGCTTCCTTTCGGGCTTTGTTACACCAGTTCGTTCGGGCAGGTT |
| pET30a-F | CTGCTAACAAAGCCCGAAAG |
| pET30a-R | TGGCACCAGACCAGAAGAAT |
| ID-Fused-F | ATGCGTCCGGCGTAGAGGATC |
| ID-Fused-R | CAGCAGCCAACTCAGCTTCCT |
| PhaC*_Ps_*_6-19_-pACY-F | CGCATAATGCTTAAGTCGAACAGAA |
| PhaC*_Ps_*_6-19_-pACY-R | GCTGCCCATGGTATATCTCCTTATT |
| sulA-F | TTTAACTTTAATAAGGAGATATACCATGTACACCAGCGGTTATGCGCACC |
| sulA-R | GATTACTTTCTGTTCGACTTAAGCATTAGTGATACAGGTTGCTGTGAATT |
| ID-sulA-PhaC*_Ps_*_6-19_-PACY-F | CGGCATACTCTGCGACAT |
| ID-sulA-PhaC*_Ps_*_6-19_-PACY-R | TACGCAAGGCGACAAGGT |

## Supplementary Table 2. ^1^H NMR and ^13^C NMR chemical shifts with corresponding assignments

| Chemical Shift (ppm) | Assignments |
| --- | --- |
| ^1^H NMR |  |
| 1.23-1.26 | -CH_3_ (LA) |
| 1.57-1.59 | -CH_3_ (PLA) |
| 3.70-3.74 | -CH- (LA) |
| 5.14-5.18 | -CH- (PLA) |
| ^13^C NMR |  |
| 16.68 | -CH_3_ |
| 68.99 | -CH- |
| 169.58 | -C=O- |

## Supplementary Figures


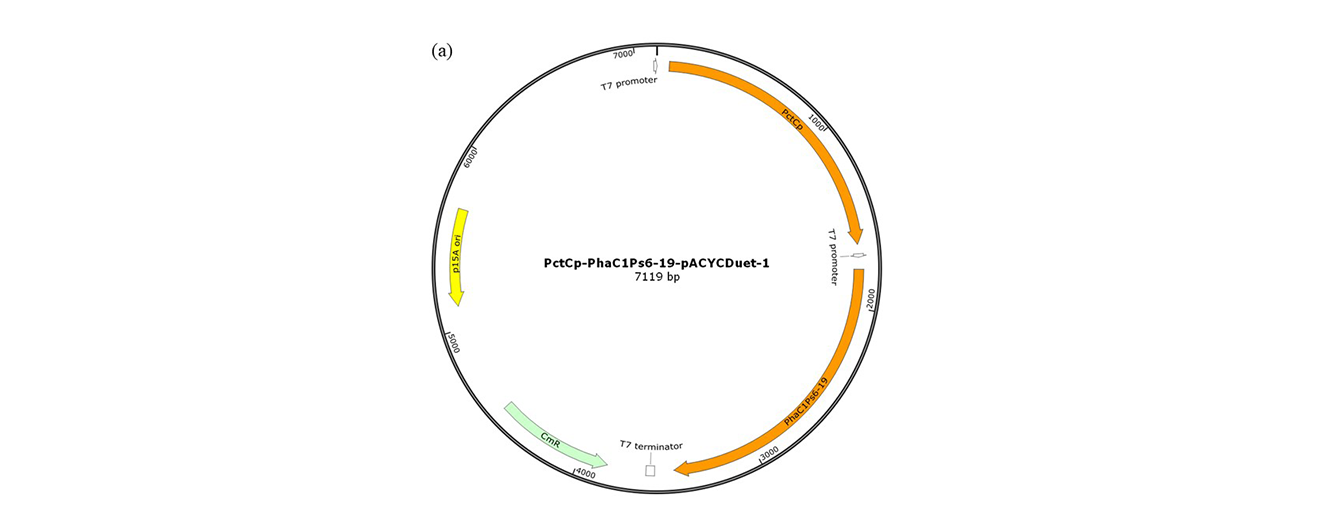

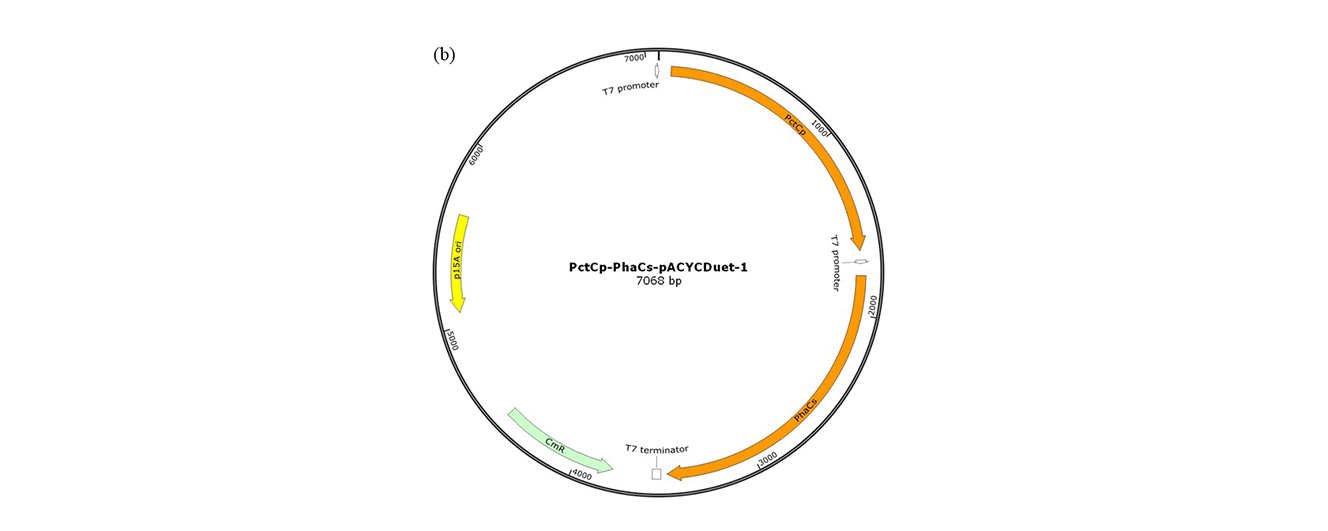

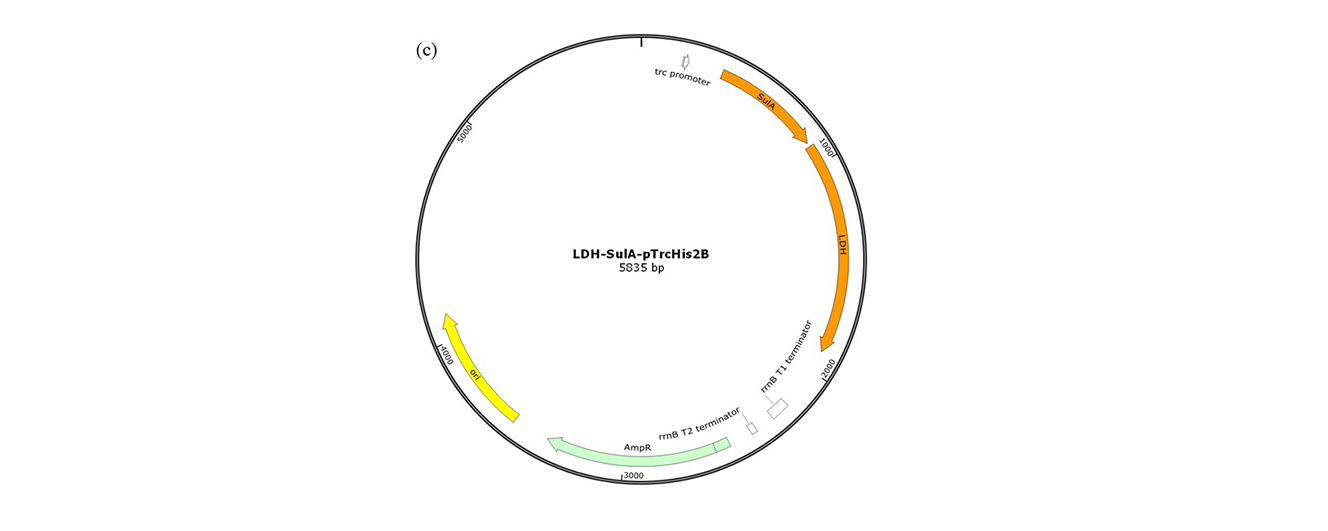


**Supplementary Figure 1.** Vector Maps of key plasmids utilized in this study. (a) Map of Pct*_Cp_*-PhaC*_Ps_*_6-19_-pACYCDuet-1. Pct from *Clostridium propionicum* containing V193A and PhaC1 from *Pseudomonas* sp. MBEL 6-19 containing quadruple mutations of E130D, S325T, S477G, and Q481K were inserted into pACYCDuet-1 vector under T7 promoters. (b) Map of Pct*_cp_*-PhaC*_Cs_*-pACYCDuet-1. Pct from *Clostridium propionicum* containing V193A and PhaC1 from *Chromobacterium* sp. USM2 were inserted into pACYCDuet-1 vector under T7 promoters; (c) Map of ldhA-sulA-pTrcHis2B. LdhA from *E. coli* BL21(DE3) and SulA from *E. coli* str. K-12 were inserted into pTrcHis2B vector under trc promoter.


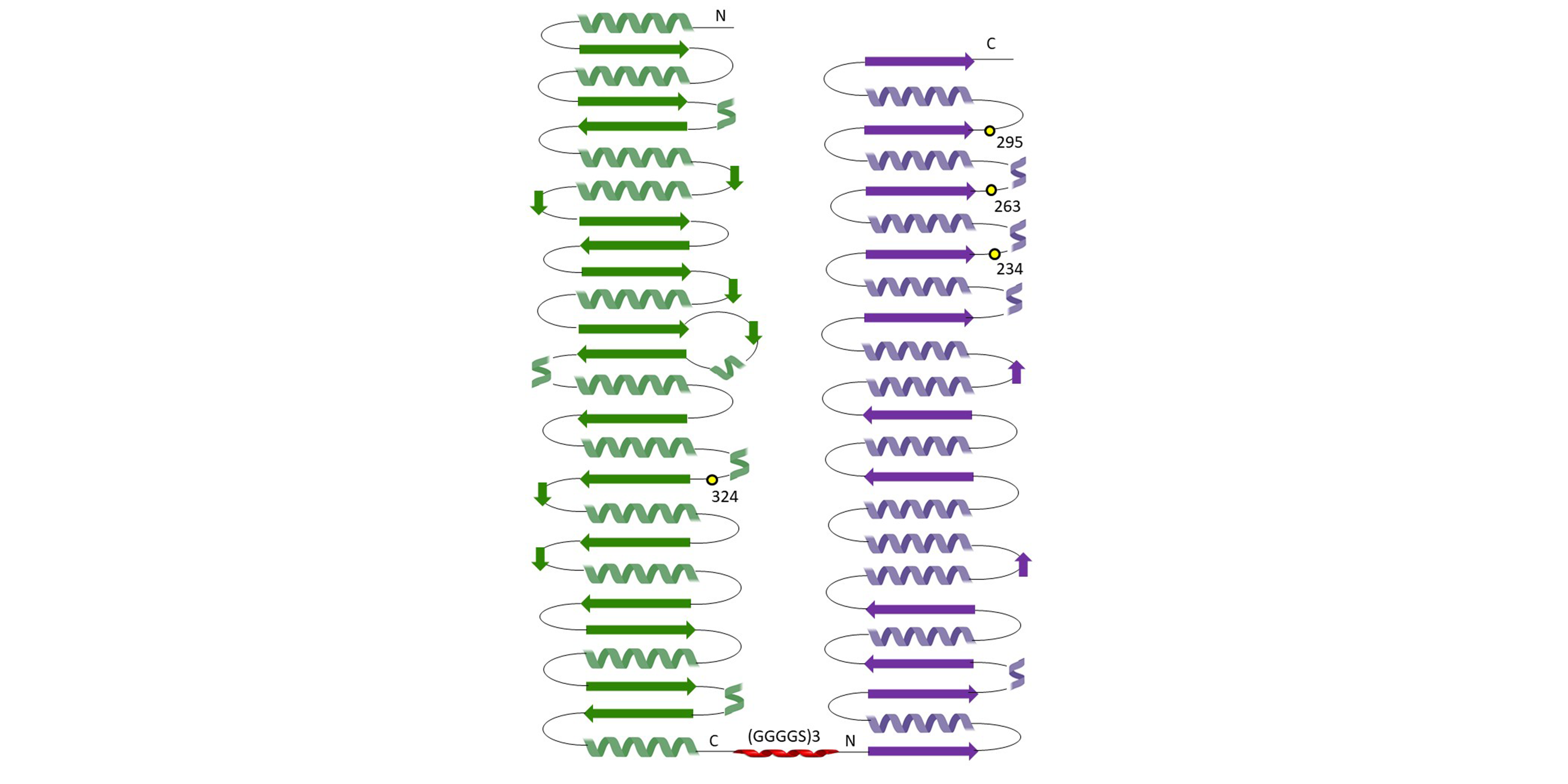


**Supplementary Figure 2.** Topology diagram of the fused enzyme of Pct*_Cp_* /LdhA. Left part presents the domain of Pct*_Cp_* (propionyl-CoA transferase from *Clostridium propionicum* DSM 1682) and the right part presents the domain of LdhA (lactate dehydrogenase from *E. coli* BL21). A flexible linker (Gly-Gly-Gly-Gly-Ser)_3_ ligates Pct*_Cp_* with LdhA. The position of active residues of the fused enzyme are indicated on the diagram.


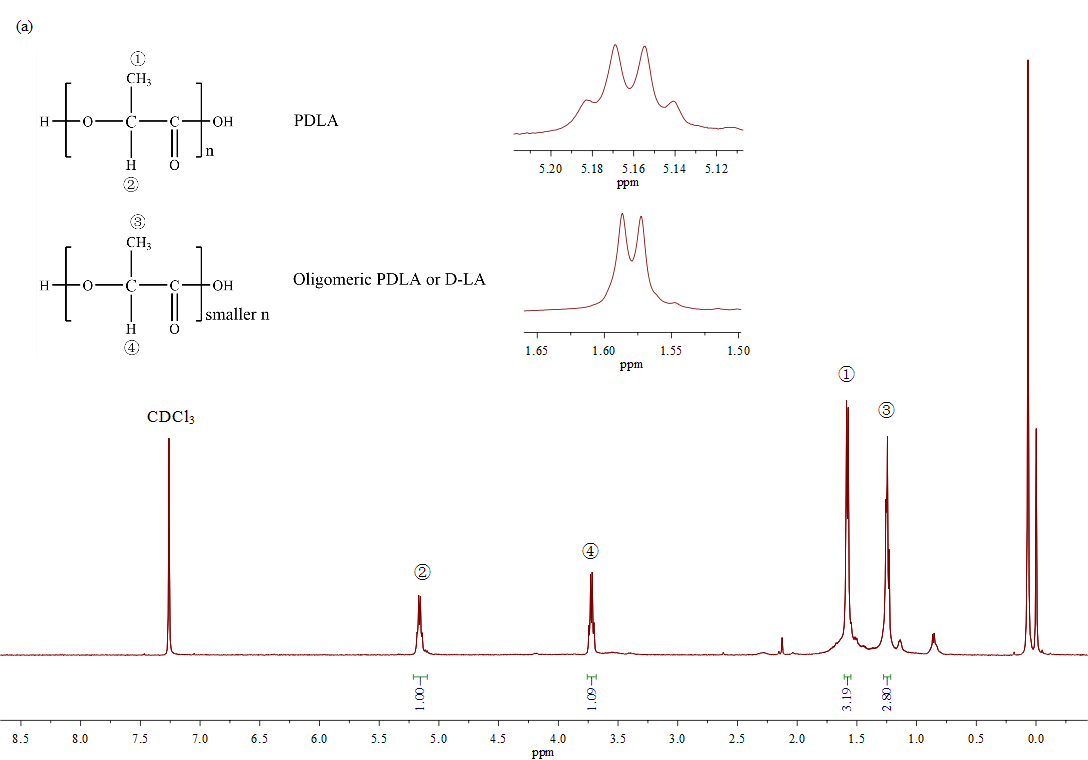


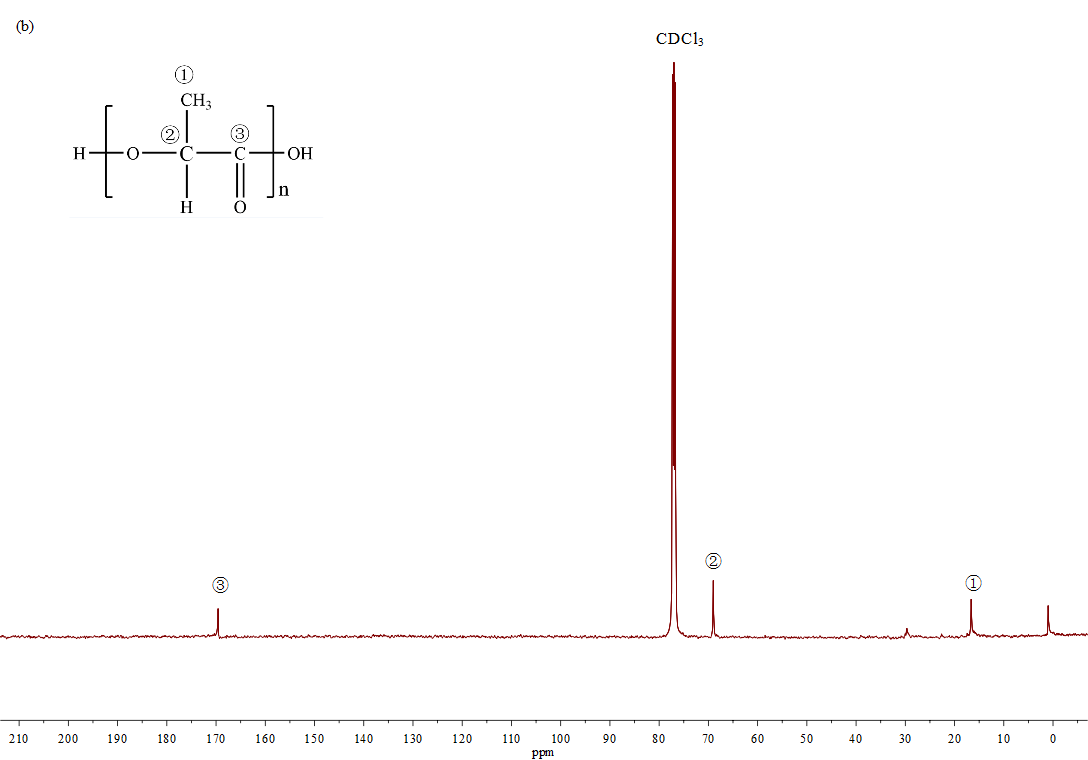


**Supplementary Figure 3**. ^1^H and ^13^C NMR assay of PLA prepared in this study. (a) ^1^H NMR spectrum of PLA. The spectrum of H doublet for methyl protons (–CH_3_) of PLA was assigned at 1.58 ppm, the quartet in the main chain for methine proton (–CH) of PLA was assigned at 5.17 ppm. Signals at about 1.25 and 3.72 ppm were assigned to methyl protons and methine proton of oligomeric PLA or D-LA monomer respectively. (b) ^13^C NMR spectrum of PLA. The carbonyl carbon is assigned at 169.58 ppm, the methine carbon (–CH) and methyl carbon (–CH_3_) of PLA are assigned at 68.99 ppm and 16.68 ppm respectively.
